# Neutron Imaging and Learning Algorithms: New Perspectives in Cultural Heritage Applications

**DOI:** 10.3390/jimaging8100284

**Published:** 2022-10-14

**Authors:** Claudia Scatigno, Giulia Festa

**Affiliations:** CREF—Museo Storico della Fisica e Centro Studi e Ricerche Enrico Fermi, Via Panisperna 89a, 00189 Rome, Italy

**Keywords:** data analysis, imaging, cultural heritage, Deep Learning, convolutional neural networks, segmentation

## Abstract

Recently, learning algorithms such as Convolutional Neural Networks have been successfully applied in different stages of data processing from the acquisition to the data analysis in the imaging context. The aim of these algorithms is the dimensionality of data reduction and the computational effort, to find benchmarks and extract features, to improve the resolution, and reproducibility performances of the imaging data. Currently, no Neutron Imaging combined with learning algorithms was applied on cultural heritage domain, but future applications could help to solve challenges of this research field. Here, a review of pioneering works to exploit the use of Machine Learning and Deep Learning models applied to X-ray imaging and Neutron Imaging data processing is reported, spanning from biomedicine, microbiology, and materials science to give new perspectives on future cultural heritage applications.

## 1. State-of-the-Art in Imaging and in Machine and Deep Learning

### 1.1. Imaging Techniques

Imaging is a powerful tool for the presentation of multi-dimensional and multi-parameter data. After data acquisition and processing, volume and surface rendering can be employed for data visualization and quantitative analyses. Imaging is used in various applications, such as engineering, remote sensing, medicine, forensic studies, and materials science [1]. The acquired dataset can be pre-processed for optimization purposes as a function of the specific setup and scope of the study. Imaging techniques produce a large amount of data, and the data analysis requires a large computational effort. Moreover, to obtain the best quality of the data, the image noise reduction is an important factor. Usually, different approaches are applied according to the characteristics of the specific sensors and the setup used for the acquisition process. Signal-to-noise ratio and resolution improvement are recently assessed through classes of Machine Learning techniques, such as Deep Learning [2].

### 1.2. Neutron Imaging and Challenges

Neutron Imaging (NI), such as radiography and tomography, are testing methods which can be used to determine the inner structure of the investigated objects. The study of the morphology and internal volumes is useful for the determination of the manufacturing processes, provenance, dating, and information about the state of conservation and restoration in the cultural heritage field [3,4,5,6]. The NI techniques exploit the interaction of the incoming neutron beam with the object under investigation. The neutron beam is registered by a position-sensitive detector after the interaction with the object. The detector system registers the information pixel-wise, while the intensity per pixel is separated into grey values, commonly in a 16-bit format, enabling to distinguish more than 65,000 intensity variations and converting into grey-scale values. This digital format allows for more advanced analysis methods such as tomography, real-time imaging, energy-selective and diffractive imaging, and grating interferometry [7,8,9]. These methods convert radiographic data into compressed information with the help of dedicated software tools. Due to the interaction processes of neutrons with the matter, they are considered an ideal probe for the non-destructive and non-invasive investigation of cultural heritage objects [3,4,5,6,7,8,9,10,11,12,13]. Indeed, neutrons interact with the atomic nuclei with a penetration depth of incident beam that is a function of the sample: for metal alloys, pottery, and stones they access the bulk of the objects without substantial attenuation while they are highly sensitive to light elements such as hydrogen [3,4,5,6,7,8,9,10,11,12,13,14,15,16,17,18]. Significant progress has been made regarding spatial and temporal resolutions, achieved in different ways such as the optimization of the optical camera that collects the signal from the scintillator screen [19] and the use of axisymmetric grazing-incidence focusing mirrors [20,21], transforming pinhole cameras into microscopes. In the framework of cultural heritage, an important issue to consider is the activation of the irradiated samples and the deactivation time that is related to the lifetime of the compound nuclei, particularly for metallic objects [3]. Indeed, the ancient objects preserved at museums must be returned within a short period with deadlines already defined in the experimental design step, and the activation parameters need to be under control. For this reason, a balance between the acquisition time has to be taken during the data acquisition phase: long acquisitions guarantee a good image quality but could dramatically activate the samples. When a sample is irradiated by a neutron beam, nuclear reactions occur such as neutron absorption, and a compound nucleus is formed producing an induced activity that is a function of the kind of isotopes and their abundance in the sample, the neutron capture cross-sections, and isotopes half-life. The latter needs to be in the order of seconds to minutes for short-lived isotopes, otherwise, the samples risk remaining active for a long time, such as in the case of the antimony (Sb^122^ and Sb^124^) with a half-life of the order of months. To avoid these activation problems, preliminary measurements via portable techniques such as X-ray fluorescence or infrared spectroscopies are carried out before moving the objects at the neutron facilities to have an idea about the composition for the prediction of the deactivation time after the neutron irradiation. These portable techniques give us only surface information and for non-homogeneous objects, eventual activation due to materials inside the ancient object, such as a sealed ceramic vase, is not predictable. For this reason, as a first step of the NI experiments, some short radiographies to check the activation of the sample are generally carried out before starting the complete tomographic scan.

### 1.3. Neutron Imaging Optimization

**Figure 1:**
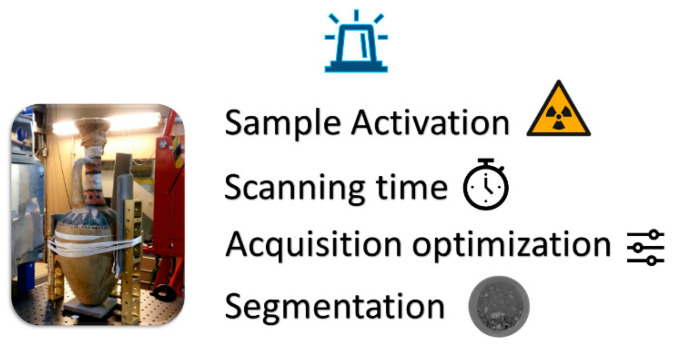
Overview of the main in Neutron Imaging applied to cultural heritage.

To obtain good NI data, quality improvements to the radiographic images are developed in previous works thanks to specific algorithms to find the best possible settings to the detriment of phase sensitivity and spatial resolution [22]. The data acquisition could be optimized through experimental procedures related to the beam characteristics and collimation, optical and detection systems, and secondary effects such as multiple scattered neutrons, and γ radiation background at the beamlines [23,24,25]. Indeed, the neutron source characteristics, such as the neutron fluence rate, could have statistical fluctuations in terms of time and space, which gives rise to the Gaussian noise, or mixed Poisson–Gaussian noise, which can affect the image quality [23]. In the data analysis process, one of the challenges in cultural heritage applications is the segmentation of the internal regions because the objects are generally composed of different materials where the edges are not sharp due to corrosion and degradation processes, or the penetration of one phase in another is very common, such as the case of the liquid vase content in the ceramic or stone matrix (Figure 1).

### 1.4. Neutron Imaging and Deep Learning

Recently, Deep Learning techniques are applied in the NI context to address the sensitivity-resolution trade problem [23]. The latter is based on a set of algorithms that attempt to model high-level abstractions in data by using model architectures, with unsupervised or semi-supervised feature learning and hierarchical feature extractions [26]. Among them, the neural network-based prediction methods, such as Convolutional Neural Networks (CNNs), are recent and are able to reduce the data acquisition time without compromising the resolution of the structure characterization to improve the temporal resolution of the time-dependent measurement [26,27]. CNNs are particularly suitable for data processing and data analysis, i.e., for object recognition, object detection, scene recognition, semantic segmentation [28,29,30], action recognition, object tracking, and many other tasks. Furthermore, Spiking Neural Networks (SNNs), are deployed on neuromorphic computing hardware which demonstrates an ultra-low power consumption [31,32]. The main purpose of Machine Learning (ML) is the reduction of high-dimensional data and can be categorized into two classes: data compression and data visualization. The supervised ML, such as the K-Nearest Neighbour (KNN) algorithm and Support Vector Regression (SVR), can reduce data and compare the training data with the predicted data by regression models, in order to attribute and validate unlabelled datasets. These techniques could be used as a selective method to extract the more significant data useful to reconstruct the image, i.e., selecting the data identified as Singular Vector (SV) belonging to the class of separation. These techniques are successfully applied in other fields [33,34] and could be employed in the cultural heritage framework. They are used to denoise the reconstruction images [33], improve their quality [33], and for optimization processes [35].

Here, a review of pioneering works to exploit the use of learning models applied to Neutron Imaging data processing is reported. The present work aims to provide an overview of the Machine Learning and Deep Learning approaches used at different levels of data imaging analysis and in different sectors that could be useful in the cultural heritage domain. Due to the similarities between X-rays, magnetic resonance imaging data, and Neutron Imaging, some applications are also reported because they can be easily translated to the neutron field.

## 2. Deep Learning Applications

Deep Learning (DL) models are successfully applied in different stages of data processing from the acquisition to the data analysis in several contexts and with different investigation techniques, spanning from X-ray computed tomography (XCT) to Neutron Imaging [23,26,33,34,35,36,37,38,39,40,41].

### 2.1. Learning Algorithms in X-ray Tomography (Biomedicine, Materials Science, and Cultural Heritage)

CNN algorithms are DL models widely used for the analysis of visual imagery, eliminating overfitting by Kernel function. They use the neural networks to detect features of images by a hierarchical structure of the data and assemble simple patterns to generate complex patterns for regularization. Amongst widespread imaging techniques, X-ray tomography is used to investigate the internal structures of a large variety of opaque samples in a non-destructive and non-invasive way. Recently, Spiking Neural Networks (SNNs) [40] are also successfully applied in medicine on magnetic resonance imaging and X-ray tomography datasets with the potential to reduce power consumption while maintaining a good performance via neuromorphic computing hardware.

The most recent applications of DL techniques exploit biomedical X-ray dataset images to detect early diagnosis [42,43,44], estimate some specific properties of complex materials [36], and characterize ancient woodblocks [45], as outlined in Table 1.

### 2.2. Learning Algorithms in Cultural Heritage Imaging

Machine Learning and Deep Learning algorithms are successfully applied to cultural heritage. In Table 2, a summary of a selection of learning methods is listed to present the recent scenarios in this field [46,47,48,49,50]. In detail: (1) ML-SVM is a supervised Machine Learning algorithm that separates data points by a hyperplane and is successfully used in handwriting recognition; (2) ML-RF is a random forest that produces outputs that are collected and combined as a tree, useful in classification and segmentation; (3) DL-CNN is a Convolutional Neural Network very suitable for image classification.

To the best of our knowledge, DL has not yet been applied extensively in NI for cultural heritage. Rather, DL was developed and has largely been applied in X-ray and magnetic resonance imaging for medical purposes [51], to automate the segmentation [52,53,54], for classification purposes [55,56,57], or, recently, applied in microscopic imaging where the potential for DL technologies is unprecedented and has still not reached its full potential [58]. Viruses, bacteria, parasites, and fungi can be monitored, investigated, and classified by the attribution of features (geometric characteristics) on microscopic images used as input in the DL models [59,60]. Details are reported in Table 3.

The techniques listed in Table 1, Table 2 and Table 3 can be applied in cultural heritage and neutron applications.

### 2.3. Deep Learning in Neutron Imaging and Future Applications in Cultural Heritage

In Neutron Imaging, Deep Learning models are successfully applied in different stages of data processing from the acquisition to the data analysis.

**Figure 2:**
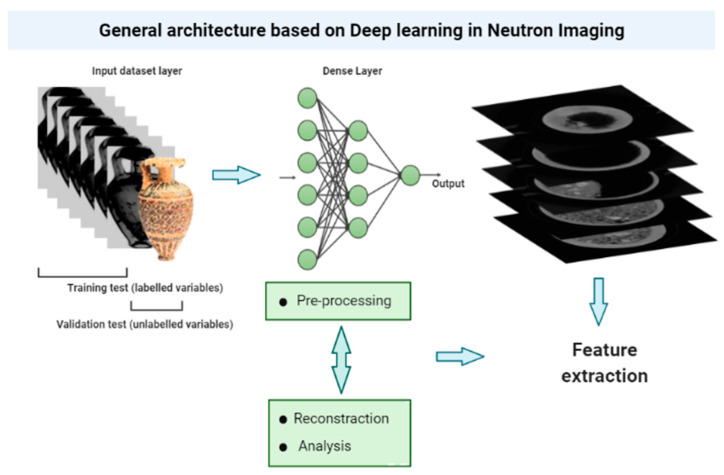
General overview of the Deep Learning architecture in Neutron Imaging applications. Training datasets that correspond to labelled variables are able to validate new datasets where the variables are still unlabelled, predicting the clustering of belonging by the similarity of their distribution along the regression line.

Figure 2 shows a general architecture of Neutron Imaging studies based on Deep Learning.

In this framework, a selection of the most relevant works is presented in Table 4 [23,25,26,38].

To optimize the prediction performance, Qiao et al. [23] applied the Gradient Magnitude Similarity Deviation (GMSD) to label the distorted images with a quality score. The labeling could be very useful in cultural heritage, where one or more categories can be classified for several purposes: authentication, conservation, and production processes identification. Micieli et al. [60], indeed, applied iterative optimization methods such as Neural Network Filtered Back-Projection (NN-FBP) to reduce the acquisition, the scan, the reconstruction time, and the amount of data storage in neutron tomography experiments. The NN-FBP model mitigates problems, i.e., artefacts, of the classical analytical reconstruction algorithm of Filtered Back-Projection. In detail, they quantitatively compared the NN-FBP, FBP, and Simultaneous Iterative Reconstruction Technique (SIRT) methods as a function of the number of projections, and applied to a set of simulated data, a phantom of Cu-CuCrZr pipe, and the real data of the pipe. The evaluation of the image quality was carried out by computing the Normalized Root Mean Square Error (NRMSE), the Structural Similarity Index (SSIM), the Feature Similarity Index (FSIM), and the Gradient Magnitude Similarity Deviation (GMSD). Tomographic scans of two similar samples were collected, generating a dataset of 1335 projections in the angular range [0°, 360°] with 30 s of exposure time per projection, approximately 11 h for a complete CT scan. The NN-FBP model reveals that the number of projections can be reduced to 223, i.e., 1/6 of the over-sampled dataset and 1/3 of the projections required by the sampling theorem (Nyquist–Shannon). The applicability in the cultural heritage context is immediate: the activation—and as a consequence, the de-activation—of the samples, in particular for metallic artefacts, is one of the main problems that need to be controlled because it is in close connection with the timing dictated by the owners, such as the museums, that require short time of permanence in the neutron facility and non-invasive and non-destructive investigations.

Lee et al. [26] successfully applied the Generative Adversarial Network (GAN) to avoid the trade-off for both high-phase sensitivity and high-resolution in interferometric applications. The GAN is a good option for image generation. It involves two neural models with conflicting objectives, one generator (G), and one discriminator (D), forcing each other to improve the output image file to again be converted back to the image file which represents the file output of the model. In detail, to obtain an image that has high-phase sensitivity as well as high-spatial resolution, a match of two images at different positions is acquired: the first one with low-contrast and high-resolution, the second one with high-contrast and low-resolution. The GAN is applied to generate the final image, a good combination of the two input images. The training dataset in ref [26] is composed of 890 image pairs; each picture has a 2000 × 2000 pixels size. The variables are connected through convolution layers (3 × 3 kernels), batch normalization, and rectified linear unit (ReLU) function. The final image is then optimized by minimizing the mean square error (MSE) between the first output image (merge of the two images) and the ground-truth image (discriminator output). The combined images from the GAN model demonstrate that the resolution and the sensitivity have been improved even if the shape is complex. In the cultural heritage context, the GAN approach could facilitate the great problem of the irregular shapes of investigated ancient objects such as pottery, archaeological objects, or ancient objects whose original morphology has changed due to degradation processes.

The last-mentioned case from Aoki et al. [38] regards the possibility to identify hidden profiles, the structure of the surfaces, and the interfaces of various materials. The morphology of the damage present in the same multi-material object is a common example. The degradation processes have a different reactivity as a function of the interfaces between the various materials. The authors [38] propose network architectures that differentiate between noisy and clean images. In detail, different blocks with different layers of convolution, batch normalization, and rectified linear units are employed for the ground truth and simulated neutron reflectometry profiles improve the efficiency of the learning process, which improves the denoising performance. In Neutron Tomography (NT), the data processing is composed of different steps: the acquisition phase at different angles, where the high number of projections of the object is acquired at different viewing angles; rotating the object around a vertical axis in the beam; the reconstruction process carried out through dedicated algorithms, such as the filtered back projection; and finally, the 3D visualization and data analysis such as segmentation. Appropriate regularization/prior-model parameters for each experiment and data acquisition are also required [61]. The acquisition time per digital image with high quality can span from a few seconds to minutes. Figure 2 shows the phases of a typical NI experiment and the possible applications of DL, from data acquisition to data analysis and visualization.

Energy-resolved Neutron Imaging has developed in recent years owing to the use of bright pulsed neutron beamline facilities at neutron spallation sources and is applied to study the composition, strain, and texture of the sample as a function of the spatial resolution [62,63,64]. Because the data are affected by noise, Machine Learning could be successfully applied to cultural heritage optimizing the acquisition time and improving the quantification results.

Table 5 shows the DL applicable in the cultural heritage domain selecting the aim and the challenge addressed with the consequent improvement [65,66].

The automatic segmentation based on U-Net can extract effective information and improve the level of material damage due to automatic learning image features. U-Net can be widely used in the cultural heritage image analysis with multiple purposes: for the identification of the cracks, i.e., to study the microstructural level of ancient concrete or mortar, to study the de-cohesions or de-adhesions of the pictorial painting on the supports, to study the stratigraphic sections segmentation, and classification of morphologies of the decay at different levels such as the degradation due to soluble salts on different supports. The U-Net network extracts deeper image features by a large number of feature channels which allows the network to propagate contextual information to higher-resolution layers [68]. Patch-CNN is particularly resolved for feature extraction. The latter, characterized by convolution algorithms, are transferred in the hierarchical fusion model to then have as output the classifications into labels [69]. Spiking Neural Networks are already tested in medical imaging and can be used in the cultural heritage context for real-time implementations for the automation of experiments. In this context, real-time identification and monitoring of the minimum number of needed radiographies collected at different angles would increase the safety of the ancient objects during the experiment, minimizing the total neutron dose and reducing activation.

## 3. Conclusions: Future Insights and Perspectives

Neutron Imaging is a powerful tool for the analysis of cultural heritage objects to obtain spatial information about the inner structure, internal morphology, and volumes that could be used to determine manufacturing processes, provenance, dating, and information about the state of conservation. In this context, the Deep Learning approach was applied in the data pre-processing phase to merge radiographs obtained by different set-ups and for the identification of the required number of radiographic images to obtain a successful reconstruction. Applications of Deep Learning in the imaging analysis phase, such as automatic segmentation, can give new ideas and perspectives. The common elements of the application of these algorithms are employed to reduce the dimensionality of the data, extract useful features to classify objects, and obtain the best images possible through optimization processes.

Cultural heritage objects are generally made of complex materials. As such, the activation of the samples and time irradiation need to be reduced as much as possible through optimization procedures to guarantee the return of ancient objects to the museum in a short time. All the experiments, indeed, need to be non-destructive and non-invasive. Moreover, these objects are characterized by a complex shape and often by a multi-material composition and get more difficult during the analysis phase. In particular, the available automatic segmentation procedures based on the grey scale could be enhanced by recognition algorithms such as semantic computational models that can classify the shape and pattern of the dataset by metric labelling. Therefore, Convolutional Neural Networks and new algorithms have been demonstrated as effective to classify the defect in metals, in concrete materials, recognize structural defects, and extract features on paintings, and can be a good candidate for future applications in Neutron Imaging. Moreover, there are new, exciting perspectives in the application of the Convolutional Neural Networks and Spiking Neural Networks architectures to solve most of the common problems in the Neutron Imaging applications in cultural heritage, from solving the problem of detection and quantification of the damaged products by automatic segmentation to estimating with high-resolution infiltration problems of ancient architectural monuments. In conclusion, Machine Learning and Deep Learning models can give new perspectives and contributions in the future for cultural heritage Neutron Imaging applications.

## Figures and Tables

**Table 1:** Three examples of Machine Learning and Deep Learning techniques applied in X-ray tomography imaging.

Method	Operation	Aim	Improvement	Limitation
CNN	Feature extraction, modeling, and fine-tuning	Early detect disease	Noise reduction	Preprocessing phase
SVR *, LR *, RF *, IGB *, CNN	Feature extraction	Predict petrophysical properties	Computation time reduction	A large calibration dataset is required
GLCM *, LBPs *, k-NN *	Feature extraction and classification	Material identification	Classification accuracies	Pretreatment data

* SVR: Support Vector Regression, LR: Linear Regression, RF: Random Forest, IGB: Improved Gradient Boosting, GLCM: grey-level co-occurrence matrix, LBPs: Local Binary Patterns, k-NN: K-Nearest Neighbor.

**Table 2:** ML and DL techniques applied in CH imaging.

Method	Dataset	Aim	Limitation	CH in NI
SVM	Digitized documents	Handwriting recognition	No replicability for other datasets	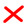
RF	Photogrammetric images	Features classification and segmentation for 3D reconstruction	Several steps	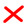
CNN	Binary profile images	Pottery classification	Resizing images	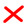

**Table 3:** Algorithms used in microbiology, with their usability and the corresponding improvement in the field.

Method	Aim	Improvement	Challenge
CNNs	Automatic detection of microorganisms	Accuracy (quantification) and speed of diagnosis	Transforming lower resolution into super-resolution images and constructing 3D images such as with fluorescence microscopy
	Virus classification (structure, size, and morphology)	Fast and cost-efficient classification	
U-Net	Segmentation of images	Detection and counting of colonies	

**Table 4:** Deep Learning algorithms used in different Neutron Imaging techniques are listed, with their usability and the corresponding Neutron Imaging in cultural heritage applicability with the improvement in the field.

Method	NI Technique	Aim	Applicability to CH-NI	Improvement
CNN-GMSD *	Radiography	Extracting features	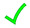	Classification of materials
NN-FBP *	Tomography	Reducing data acquisition time and data storage	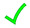	Especially for active materials
CNN-GAN *	Interferometric phase-contrast imaging	Improving resolution and the sensitivity	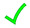	Shapes complex
pCNN, DnCNN, CAE *	Reflectometry	Extracting hidden profiles in large statistical noise	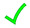	Multimaterial, different shapes

* GMSD: Gradient Magnitude Similarity Deviation, NN-FBP: Neural Network Filtered Back-Projection, GAN: Generative Adversarial Network, pCNN: Plain CNN, DnCNN: Denoising CNN. CAE: Convolutional Autoencoder.

**Table 5:** NT and NR in cultural heritage and the improvements obtained via DL algorithms.

Material Investigated	Aim	Challenge	DL Applicable	Improvement
Metallic statue	Origin, purpose, manufacturing process, provenance	Quantitative purpose (filled voxels are hardly considered)	CNNs/U-Net	Automatic counting of the voxels
	restoration and conservation purpose (distinguishing different corrosion zones)	Classification of different corrosion products	CNNs/Patch-CNN	Detection and quantification of the damaged products by automatic segmentation
Ancient concrete	Studying the microstructure, cracks, and water adsorption	Dynamics of water penetration	U-Net/SBL *	High-resolution estimation

* SBL: Sparse Bayesian Learning [67].

## Data Availability

Not applicable.
